# Supplementary material for: Dose response of running on blood biomarkers of wellness in generally healthy individuals
Source: PLoS One. 2023 Nov 15;18(11):e0293631. doi: 10.1371/journal.pone.0293631 (PMC10651037; doi:10.1371/journal.pone.0293631)
Supplement: S1 File — (PDF) [file pone.0293631.s001.pdf]

S1 Table. Number of people in each category by age group. Significant trend toward younger individuals reporting higher running volume, with more than 75% of the elite group falling between the ages of 18 and 35.

Age Group	PRO	HVAM	MVAM	LVAM	SED
18-35	53	434	1975	3452	1032
35-45	25	366	2537	3964	1431
45-55	4	218	1606	2469	1152
>55	NA	85	629	992	813

S2 Table. Full running volume vs. blood biomarker results

Biomarker	ANOVA p-value	Trend p-value	lowest mean	highest mean
**Alb**	<1e-16	<0.001	MVAM	PRO
**ALT**	<1e-16	<1e-16	SED	PRO
**AST**	<1e-16	<0.001	SED	PRO
**B12**	<0.001	<0.001	SED	PRO
**BASOS**	0.001	0.004	LVAM	PRO
**BASOS_PCT**	<0.001	0.156	SED	PRO
**Ca**	0.007	0.030	MVAM	PRO
**Chol**	<0.001	0.005	PRO	SED
**CK**	<1e-16	<1e-16	SED	PRO
**Cor**	<0.001	0.675	SED	PRO
**D**	<1e-16	0.424	SED	PRO
**DHEAS**	<0.001	<0.001	SED	PRO
**EOS**	<0.001	0.371	HVAM	SED
**EOS_PCT**	<0.001	0.137	HVAM	MVAM
**FE**	<0.001	0.119	SED	PRO
**Fer**	<1e-16	<1e-16	MVAM	SED
**Fol**	<1e-16	<0.001	SED	PRO
**FT**	<0.001	0.013	SED	PRO
**GGT**	<1e-16	<0.001	PRO	SED
**Glu**	0.087	0.184	PRO	SED
**Hb**	0.002	<0.001	MVAM	PRO
**HCT**	0.053	0.055	MVAM	PRO
**HDL**	<1e-16	<0.001	SED	PRO
**HbA1c**	<0.001	0.010	PRO	SED
**hsCRP**	<0.001	0.176	PRO	SED
**K**	<1e-16	<0.001	SED	LVAM
**LDL**	<0.001	0.006	PRO	SED
**LYMPHS**	<0.001	0.008	PRO	SED
**LYMPHS_PCT**	<1e-16	0.417	SED	PRO
**MCH**	0.197	0.077	SED	PRO
**MCHC**	<1e-16	0.276	SED	PRO
**MCV**	<0.001	<0.001	SED	PRO
**Mg**	<0.001	0.276	PRO	SED
**MONOS**	<0.001	0.175	PRO	SED
**MONOS_PCT**	<0.001	0.137	SED	LVAM
**MPV**	0.058	0.089	SED	HVAM
**Na**	<1e-16	0.622	HVAM	SED
**NEUT**	<0.001	0.007	PRO	SED
**NEUT_PCT**	<0.001	0.764	PRO	SED
**PLT**	<0.001	0.058	LVAM	SED
**RBC**	0.016	0.880	MVAM	SED
**RBC_Mg**	<0.001	0.773	PRO	SED
**RDW**	<1e-16	0.002	PRO	SED
**SHBG**	<1e-16	0.004	SED	PRO
**Tes**	<1e-16	0.675	MVAM	LVAM
**Tg**	<1e-16	<1e-16	PRO	SED
**TIBC**	<0.001	0.417	LVAM	MVAM
**TS**	<1e-16	0.298	SED	PRO
**WBC**	<1e-16	<1e-16	PRO	SED

S3 Table. 2S-MR results with BMI as the exposure and select biomarkers as outcomes

Exposure-outcome	Method	Causal estimate			
		SNP	Beta	SE	*p*
BMI-hsCRP	MR Egger	233	0.440	0.054	1.74E-14
	Weighted median	233	0.385	0.022	7.56E-71
	Inverse variance weighted	233	0.352	0.018	6.78E-84
	Simple mode	233	0.403	0.067	8.11E-09
	Weighted mode	233	0.413	0.039	9.98E-22
Test for horizontal pleiotropy: MR Egger intercept = -0.00191, SE = 0.0109, p = 0.0816					
BMI-HbA1c	MR Egger	213	0.084	0.042	4.57E-02
	Weighted median	213	0.036	0.022	9.98E-02
	Inverse variance weighted	213	0.031	0.014	3.16E-02
	Simple mode	213	0.021	0.055	7.07E-01
	Weighted mode	213	0.080	0.036	2.88E-02
Test for horizontal pleiotropy: MR Egger intercept = -0.00115, SE = 0.000855, p = 0.178					
BMI-Tg	MR Egger	223	0.302	0.065	5.45E-06
	Weighted median	223	0.227	0.029	5.83E-15
	Inverse variance weighted	223	0.203	0.022	6.79E-20
	Simple mode	223	0.216	0.085	1.20E-02
	Weighted mode	223	0.239	0.049	2.45E-06
Test for horizontal pleiotropy: MR Egger intercept = -0.00220, SE = 0.00135, p = 0.105					
BMI-HDL	MR Egger	223	-0.341	0.068	1.13E-06
	Weighted median	223	-0.255	0.031	1.24E-16
	Inverse variance weighted	223	-0.265	0.023	3.28E-30
	Simple mode	223	-0.050	0.074	5.01E-01
	Weighted mode	223	-0.285	0.045	1.73E-09
Test for horizontal pleiotropy: MR Egger intercept = -0.00169, SE = 0.00141, p = 0.234					
BMI-WBC	MR Egger	298	-0.035	0.045	4.34E-01
	Weighted median	298	0.031	0.011	6.15E-03
	Inverse variance weighted	298	0.019	0.015	2.23E-01
	Simple mode	298	0.028	0.037	4.46E-01
	Weighted mode	298	0.054	0.030	7.28E-02

Test for horizontal pleiotropy: MR Egger intercept = -0.00118, SE = 0.000921, p = 0.202					
BMI-cortisol	MR Egger	246	-0.108	0.149	4.69E-01
	Weighted median	246	-0.068	0.075	3.63E-01
	Inverse variance weighted	246	-0.014	0.049	7.80E-01
	Simple mode	246	-0.115	0.207	5.78E-01
	Weighted mode	246	-0.173	0.130	1.86E-01
Test for horizontal pleiotropy: MR Egger intercept = -0.00202, SE = 0.00301, p = 0.503					
BMI-SHBG	MR Egger	492	-6.193	1.182	2.40E-07
	Weighted median	492	-6.065	0.383	1.67E-56
	Inverse variance weighted	492	-7.084	0.445	5.67E-57
	Simple mode	492	-6.315	1.277	1.06E-06
	Weighted mode	492	-5.343	0.777	1.88E-11
Test for horizontal pleiotropy: MR Egger intercept = -0.0156, SE = 0.0191, p = 0.416					
BMI-folate	MR Egger	492	-0.009	0.052	8.69E-01
	Weighted median	492	-0.023	0.029	4.17E-01
	Inverse variance weighted	492	-0.059	0.019	2.27E-03
	Simple mode	492	-0.011	0.091	9.03E-01
	Weighted mode	492	0.025	0.052	6.36E-01
Test for horizontal pleiotropy: MR Egger intercept = -0.000891, SE = 0.000836, p = 0.288					
BMI-RDW	MR Egger	300	0.245	0.055	1.18E-05
	Weighted median	300	0.169	0.021	1.67E-15
	Inverse variance weighted	300	0.162	0.019	1.08E-17
	Simple mode	300	0.192	0.060	1.53E-03
	Weighted mode	300	0.211	0.039	1.03E-07
Test for horizontal pleiotropy: MR Egger intercept = -0.00184, SE = 0.001134, p = 0.107					
BMI-Glu	MR Egger	64	0.107	0.035	3.05E-03
	Weighted median	64	0.088	0.019	5.11E-06
	Inverse variance weighted	64	0.093	0.015	3.08E-10
	Simple mode	64	0.094	0.035	9.83E-03
	Weighted mode	64	0.089	0.022	1.87E-04
Test for horizontal pleiotropy: MR Egger intercept = -0.0003952, SE = 0.00084453, p = 0.6415					
BMI-Fer	MR Egger	206	0.008	0.112	9.41E-01

	Weighted median	206	0.108	0.057	5.91E-02
	Inverse variance weighted	206	0.132	0.039	6.44E-04
	Simple mode	206	0.196	0.180	2.77E-01
	Weighted mode	206	0.012	0.114	9.17E-01
Test for horizontal pleiotropy: MR Egger intercept = 0.002695, SE = 0.002299, p = 0.242					
BMI-Tes	MR Egger	205	-0.262	0.112	2.05E-02
	Weighted median	205	-0.337	0.060	1.82E-08
	Inverse variance weighted	205	-0.147	0.041	3.61E-04
	Simple mode	205	-0.559	0.153	3.25E-04
	Weighted mode	205	-0.440	0.091	2.71E-06
Test for horizontal pleiotropy: MR Egger intercept = 0.002656, SE = 0.002407, p = 0.271					
BMI-Baso	MR Egger	304	-0.006	0.038	8.81E-01
	Weighted median	304	-0.036	0.018	4.70E-02
	Inverse variance weighted	304	-0.009	0.013	4.90E-01
	Simple mode	304	-0.069	0.055	2.05E-01
	Weighted mode	304	-0.059	0.042	1.63E-01
Test for horizontal pleiotropy: MR Egger intercept = -7.13E-05, SE = 0.000785, p = 0.928					
BMI-Mono	MR Egger	298	-0.097	0.045	3.07E-02
	Weighted median	298	-0.015	0.011	1.82E-01
	Inverse variance weighted	298	-0.018	0.015	2.37E-01
	Simple mode	298	-0.004	0.035	9.18E-01
	Weighted mode	298	-0.016	0.023	4.84E-01
Test for horizontal pleiotropy: MR Egger intercept = 0.001740, SE = 0.000926404, p = 0.0613					
BMI-MCV	MR Egger	300	-0.108	0.147	4.62E-01
	Weighted median	300	-0.141	0.079	7.48E-02
	Inverse variance weighted	300	-0.087	0.049	7.42E-02
	Simple mode	300	-0.097	0.229	6.71E-01
	Weighted mode	300	-0.147	0.158	3.53E-01
Test for horizontal pleiotropy: MR Egger intercept = 0.000459, SE = 0.003011, p = 0.879					
BMI-Lymph	MR Egger	298	-0.076	0.048	1.11E-01
	Weighted median	298	-0.026	0.012	3.15E-02
	Inverse variance weighted	298	0.020	0.016	2.20E-01
	Simple mode	298	-0.028	0.038	4.64E-01
	Weighted mode	298	-0.048	0.022	3.20E-02
Test for horizontal pleiotropy: MR Egger intercept =0.002111, SE = 0.0009811, p = 0.032161341					

S4 Table. 2S-MR results with BMI with biomarkers as exposures and BMI as outcome to assess reverse causality

id.exposure	id.outcome	outcome	exposure	method	nsnp	b	se	pval
**ebi-a-GCST004631**	ukb-a-248	Body mass index (BMI) || id:ukb-a-248	Basophil percentage of white cells || id:ebi-a-GCST004631	MR Egger	55	0.02850274	0.03058163	0.35555179
**ebi-a-GCST004631**	ukb-a-248	Body mass index (BMI) || id:ukb-a-248	Basophil percentage of white cells || id:ebi-a-GCST004631	Weighted median	55	0.01115946	0.01802141	0.53576264
**ebi-a-GCST004631**	ukb-a-248	Body mass index (BMI) || id:ukb-a-248	Basophil percentage of white cells || id:ebi-a-GCST004631	Inverse variance weighted	55	-0.0133255	0.01548404	0.38946137
**ebi-a-GCST004631**	ukb-a-248	Body mass index (BMI) || id:ukb-a-248	Basophil percentage of white cells || id:ebi-a-GCST004631	Simple mode	55	0.00553647	0.03733532	0.88266604
**ebi-a-GCST004631**	ukb-a-248	Body mass index (BMI) || id:ukb-a-248	Basophil percentage of white cells || id:ebi-a-GCST004631	Weighted mode	55	-0.0005482	0.02054445	0.97881084
**id.exposure**	id.outcome	outcome	exposure	method	nsnp	b	se	pval
**ieu-a-1012**	ukb-a-248	Body mass index (BMI) || id:ukb-a-248	Plasma cortisol || id:ieu-a-1012	Wald ratio	1	-0.0162841	0.0285215	0.56803913
**id.exposure**	id.outcome	outcome	exposure	method	nsnp	b	se	pval
**ieu-a-1050**	ukb-a-248	Body mass index (BMI) || id:ukb-a-248	Ferritin || id:ieu-a-1050	MR Egger	4	-0.0692185	0.02954547	0.14388806
**ieu-a-1050**	ukb-a-248	Body mass index (BMI) || id:ukb-a-248	Ferritin || id:ieu-a-1050	Weighted median	4	-0.0458501	0.01819113	0.01171994
**ieu-a-1050**	ukb-a-248	Body mass index (BMI) || id:ukb-a-248	Ferritin || id:ieu-a-1050	Inverse variance weighted	4	-0.0401901	0.01571805	0.01055975
**ieu-a-1050**	ukb-a-248	Body mass index (BMI) || id:ukb-a-248	Ferritin || id:ieu-a-1050	Simple mode	4	-0.040832	0.02570683	0.21040991
**ieu-a-1050**	ukb-a-248	Body mass index (BMI) || id:ukb-a-248	Ferritin || id:ieu-a-1050	Weighted mode	4	-0.0508659	0.01904314	0.07562034

id.exposure	id.outcome	outcome	exposure	method	nsnp	b	se	pval
**ukb-b-11349**	ieu-b-40	body mass index || id:ieu-b-40	Folate || id:ukb-b-11349	Wald ratio	1	0.04546597	0.05883832	0.4396838
**id.exposure**	id.outcome	outcome	exposure	method	nsnp	b	se	pval
**ieu-b-114**	ukb-a-248	Body mass index (BMI) || id:ukb-a-248	Fasting glucose || id:ieu-b-114	MR Egger	30	-0.0453952	0.11342853	0.69203895
**ieu-b-114**	ukb-a-248	Body mass index (BMI) || id:ukb-a-248	Fasting glucose || id:ieu-b-114	Weighted median	30	0.00266784	0.03188398	0.93331616
**ieu-b-114**	ukb-a-248	Body mass index (BMI) || id:ukb-a-248	Fasting glucose || id:ieu-b-114	Inverse variance weighted	30	-0.0341858	0.05296448	0.51863721
**ieu-b-114**	ukb-a-248	Body mass index (BMI) || id:ukb-a-248	Fasting glucose || id:ieu-b-114	Simple mode	30	-0.01322	0.06706873	0.84511555
**ieu-b-114**	ukb-a-248	Body mass index (BMI) || id:ukb-a-248	Fasting glucose || id:ieu-b-114	Weighted mode	30	0.00016512	0.02915844	0.99552048
**id.exposure**	id.outcome	outcome	exposure	method	nsnp	b	se	pval
**ieu-a-270**	ukb-a-248	Body mass index (BMI) || id:ukb-a-248	Haemoglobin concentration || id:ieu-a-270	MR Egger	15	0.00144021	0.07587258	0.98514372
**ieu-a-270**	ukb-a-248	Body mass index (BMI) || id:ukb-a-248	Haemoglobin concentration || id:ieu-a-270	Weighted median	15	0.01307681	0.02023195	0.51805628
**ieu-a-270**	ukb-a-248	Body mass index (BMI) || id:ukb-a-248	Haemoglobin concentration || id:ieu-a-270	Inverse variance weighted	15	-0.0334432	0.02660806	0.20879683
**ieu-a-270**	ukb-a-248	Body mass index (BMI) || id:ukb-a-248	Haemoglobin concentration || id:ieu-a-270	Simple mode	15	-0.1129022	0.05448673	0.05720502
**ieu-a-270**	ukb-a-248	Body mass index (BMI) || id:ukb-a-248	Haemoglobin concentration || id:ieu-a-270	Weighted mode	15	0.01648048	0.02138074	0.45363255

<table>
<tr><th>id.exposure</th><th>id.outcome</th><th>outcome</th><th>exposure</th><th>method</th><th>nsnp</th><th>b</th><th>se</th><th>pval</th></tr>
<tr><td>ieu-b-103</td><td>ukb-a-248</td><td>Body mass index (BMI) || id:ukb-a-248</td><td>HbA1C || id:ieu-b-103</td><td>MR Egger</td><td>11</td><td>0.01268313</td><td>0.08396</td><td>0.88325885</td></tr>
<tr><td>ieu-b-103</td><td>ukb-a-248</td><td>Body mass index (BMI) || id:ukb-a-248</td><td>HbA1C || id:ieu-b-103</td><td>Weighted median</td><td>11</td><td>-0.0069654</td><td>0.03248061</td><td>0.83019839</td></tr>
<tr><td>ieu-b-103</td><td>ukb-a-248</td><td>Body mass index (BMI) || id:ukb-a-248</td><td>HbA1C || id:ieu-b-103</td><td>Inverse variance weighted</td><td>11</td><td>0.0283815</td><td>0.03446064</td><td>0.41017145</td></tr>
<tr><td>ieu-b-103</td><td>ukb-a-248</td><td>Body mass index (BMI) || id:ukb-a-248</td><td>HbA1C || id:ieu-b-103</td><td>Simple mode</td><td>11</td><td>-0.0257779</td><td>0.06293863</td><td>0.69075483</td></tr>
<tr><td>ieu-b-103</td><td>ukb-a-248</td><td>Body mass index (BMI) || id:ukb-a-248</td><td>HbA1C || id:ieu-b-103</td><td>Weighted mode</td><td>11</td><td>-0.0208929</td><td>0.03914067</td><td>0.60514756</td></tr>
<tr><td>id.exposure</td><td>id.outcome</td><td>outcome</td><td>exposure</td><td>method</td><td>nsnp</td><td>b</td><td>se</td><td>pval</td></tr>
<tr><td>ieu-a-275</td><td>ukb-a-248</td><td>Body mass index (BMI) || id:ukb-a-248</td><td>Red blood cell count || id:ieu-a-275</td><td>MR Egger</td><td>26</td><td>0.26135491</td><td>0.14952437</td><td>0.09326378</td></tr>
<tr><td>ieu-a-275</td><td>ukb-a-248</td><td>Body mass index (BMI) || id:ukb-a-248</td><td>Red blood cell count || id:ieu-a-275</td><td>Weighted median</td><td>26</td><td>0.08455861</td><td>0.04638846</td><td>0.06832803</td></tr>
<tr><td>ieu-a-275</td><td>ukb-a-248</td><td>Body mass index (BMI) || id:ukb-a-248</td><td>Red blood cell count || id:ieu-a-275</td><td>Inverse variance weighted</td><td>26</td><td>-0.0072778</td><td>0.05705404</td><td>0.89849796</td></tr>
<tr><td>ieu-a-275</td><td>ukb-a-248</td><td>Body mass index (BMI) || id:ukb-a-248</td><td>Red blood cell count || id:ieu-a-275</td><td>Simple mode</td><td>26</td><td>0.09054068</td><td>0.07897524</td><td>0.26246646</td></tr>
<tr><td>ieu-a-275</td><td>ukb-a-248</td><td>Body mass index (BMI) || id:ukb-a-248</td><td>Red blood cell count || id:ieu-a-275</td><td>Weighted mode</td><td>26</td><td>0.09962869</td><td>0.04750758</td><td>0.04626112</td></tr>
</table>

id.exposure	id.outcome	outcome	exposure	method	nsnp	b	se	pval
**ebi-a-GCST005068**	ieu-b-40	body mass index || id:ieu-b-40	LDL cholesterol || id:ebi-a-GCST005068	MR Egger	4	0.01267769	0.05816618	0.84767998
**ebi-a-GCST005068**	ieu-b-40	body mass index || id:ieu-b-40	LDL cholesterol || id:ebi-a-GCST005068	Weighted median	4	-0.0332107	0.01134854	0.00342875
**ebi-a-GCST005068**	ieu-b-40	body mass index || id:ieu-b-40	LDL cholesterol || id:ebi-a-GCST005068	Inverse variance weighted	4	-0.0320938	0.00863712	0.00020257
**ebi-a-GCST005068**	ieu-b-40	body mass index || id:ieu-b-40	LDL cholesterol || id:ebi-a-GCST005068	Simple mode	4	-0.036639	0.01673254	0.11628833
**ebi-a-GCST005068**	ieu-b-40	body mass index || id:ieu-b-40	LDL cholesterol || id:ebi-a-GCST005068	Weighted mode	4	-0.034971	0.01482963	0.09956398
**id.exposure**	id.outcome	outcome	exposure	method	nsnp	b	se	pval
**ebi-a-GCST90002336**	ukb-a-248	Body mass index (BMI) || id:ukb-a-248	Mean corpuscular volume || id:ebi-a-GCST90002336	MR Egger	9	0.01627788	0.03256415	0.63249338
**ebi-a-GCST90002336**	ukb-a-248	Body mass index (BMI) || id:ukb-a-248	Mean corpuscular volume || id:ebi-a-GCST90002336	Weighted median	9	-0.0169769	0.01096864	0.12167813
**ebi-a-GCST90002336**	ukb-a-248	Body mass index (BMI) || id:ukb-a-248	Mean corpuscular volume || id:ebi-a-GCST90002336	Inverse variance weighted	9	-0.0212777	0.01246612	0.08785201
**ebi-a-GCST90002336**	ukb-a-248	Body mass index (BMI) || id:ukb-a-248	Mean corpuscular volume || id:ebi-a-GCST90002336	Simple mode	9	-0.0220168	0.01637922	0.21575632
**ebi-a-GCST90002336**	ukb-a-248	Body mass index (BMI) || id:ukb-a-248	Mean corpuscular volume || id:ebi-a-GCST90002336	Weighted mode	9	-0.0231087	0.01392193	0.13552035

id.exposure	id.outcome	outcome	exposure	method	nsnp	b	se	pval
**ieu-a-1008**	ukb-a-248	Body mass index (BMI) || id:ukb-a-248	Platelet count || id:ieu-a-1008	MR Egger	32	-0.0006727	0.00070143	0.34517459
**ieu-a-1008**	ukb-a-248	Body mass index (BMI) || id:ukb-a-248	Platelet count || id:ieu-a-1008	Weighted median	32	0.00013472	0.00021428	0.52954228
**ieu-a-1008**	ukb-a-248	Body mass index (BMI) || id:ukb-a-248	Platelet count || id:ieu-a-1008	Inverse variance weighted	32	-0.0003268	0.00024878	0.18895678
**ieu-a-1008**	ukb-a-248	Body mass index (BMI) || id:ukb-a-248	Platelet count || id:ieu-a-1008	Simple mode	32	9.63E-05	0.00034761	0.78352525
**ieu-a-1008**	ukb-a-248	Body mass index (BMI) || id:ukb-a-248	Platelet count || id:ieu-a-1008	Weighted mode	32	0.00011659	0.00024456	0.63688891
**id.exposure**	id.outcome	outcome	exposure	method	nsnp	b	se	pval
**ieu-a-275**	ukb-a-248	Body mass index (BMI) || id:ukb-a-248	Red blood cell count || id:ieu-a-275	MR Egger	26	0.26135491	0.14952437	0.09326378
**ieu-a-275**	ukb-a-248	Body mass index (BMI) || id:ukb-a-248	Red blood cell count || id:ieu-a-275	Weighted median	26	0.08455861	0.04591029	0.06550111
**ieu-a-275**	ukb-a-248	Body mass index (BMI) || id:ukb-a-248	Red blood cell count || id:ieu-a-275	Inverse variance weighted	26	-0.0072778	0.05705404	0.89849796
**ieu-a-275**	ukb-a-248	Body mass index (BMI) || id:ukb-a-248	Red blood cell count || id:ieu-a-275	Simple mode	26	0.09054068	0.07653474	0.24793721
**ieu-a-275**	ukb-a-248	Body mass index (BMI) || id:ukb-a-248	Red blood cell count || id:ieu-a-275	Weighted mode	26	0.09962869	0.04605998	0.04030183

id.exposure	id.outcome	outcome	exposure	method	nsnp	b	se	pval
**ebi-a-GCST006804**	ukb-a-248	Body mass index (BMI) || id:ukb-a-248	Red cell distribution width || id:ebi-a-GCST006804	MR Egger	122	-0.0341912	0.03423066	0.31987855
**ebi-a-GCST006804**	ukb-a-248	Body mass index (BMI) || id:ukb-a-248	Red cell distribution width || id:ebi-a-GCST006804	Weighted median	122	0.01563428	0.00970492	0.10718764
**ebi-a-GCST006804**	ukb-a-248	Body mass index (BMI) || id:ukb-a-248	Red cell distribution width || id:ebi-a-GCST006804	Inverse variance weighted	122	0.0300081	0.01723486	0.08166104
**ebi-a-GCST006804**	ukb-a-248	Body mass index (BMI) || id:ukb-a-248	Red cell distribution width || id:ebi-a-GCST006804	Simple mode	122	0.01857783	0.02026892	0.3611926
**ebi-a-GCST006804**	ukb-a-248	Body mass index (BMI) || id:ukb-a-248	Red cell distribution width || id:ebi-a-GCST006804	Weighted mode	122	0.01128028	0.01217303	0.35594689

id.exposure	id.outcome	outcome	exposure	method	nsnp	b	se	pval
**ieu-a-302**	ukb-a-248	Body mass index (BMI) || id:ukb-a-248	Triglycerides || id:ieu-a-302	MR Egger	55	-0.0445751	0.03601334	0.22126815
**ieu-a-302**	ukb-a-248	Body mass index (BMI) || id:ukb-a-248	Triglycerides || id:ieu-a-302	Weighted median	55	-0.0315603	0.01590831	0.04726817
**ieu-a-302**	ukb-a-248	Body mass index (BMI) || id:ukb-a-248	Triglycerides || id:ieu-a-302	Inverse variance weighted	55	-0.0214287	0.02233545	0.33735629
**ieu-a-302**	ukb-a-248	Body mass index (BMI) || id:ukb-a-248	Triglycerides || id:ieu-a-302	Simple mode	55	-0.0456892	0.02952211	0.1275531
**ieu-a-302**	ukb-a-248	Body mass index (BMI) || id:ukb-a-248	Triglycerides || id:ieu-a-302	Weighted mode	55	-0.0297723	0.01209893	0.01709375

id.exposure	id.outcome	outcome	exposure	method	nsnp	b	se	pval
**ieu-b-30**	ukb-a-248	Body mass index (BMI) || id:ukb-a-248	white blood cell count || id:ieu-b-30	MR Egger	475	-0.0347487	0.02445633	0.15602056
**ieu-b-30**	ukb-a-248	Body mass index (BMI) || id:ukb-a-248	white blood cell count || id:ieu-b-30	Weighted median	475	-0.0307482	0.01204555	0.01069025
**ieu-b-30**	ukb-a-248	Body mass index (BMI) || id:ukb-a-248	white blood cell count || id:ieu-b-30	Inverse variance weighted	475	-0.040535	0.01168163	0.0005205
**ieu-b-30**	ukb-a-248	Body mass index (BMI) || id:ukb-a-248	white blood cell count || id:ieu-b-30	Simple mode	475	0.00133145	0.03782675	0.97193623
**ieu-b-30**	ukb-a-248	Body mass index (BMI) || id:ukb-a-248	white blood cell count || id:ieu-b-30	Weighted mode	475	-0.0184754	0.02085621	0.37614876

id.exposure	id.outcome	outcome	exposure	method	nsnp	b	se	pval
**ukb-d-30830_raw**	ieu-b-40	body mass index || id:ieu-b-40	SHBG || id:ukb-d-30830_raw	MR Egger	135	0.00121356	0.00180979	0.50366715
**ukb-d-30830_raw**	ieu-b-40	body mass index || id:ieu-b-40	SHBG || id:ukb-d-30830_raw	Weighted median	135	0.00102542	0.00037386	0.00609279
**ukb-d-30830_raw**	ieu-b-40	body mass index || id:ieu-b-40	SHBG || id:ukb-d-30830_raw	Inverse variance weighted	135	-0.002129	0.001153	0.06482013
**ukb-d-30830_raw**	ieu-b-40	body mass index || id:ieu-b-40	SHBG || id:ukb-d-30830_raw	Simple mode	135	-0.0011256	0.00124723	0.36841147

S5 Table. 2S-MR results with vigorous physical activity as exposure and blood biomarkers as outcomes

id.exposure	id.outcome	outcome	exposure	method	nsnp	b	se	pval
**ebi-a-GCST006098**	ebi-a-GCST005068	LDL cholesterol || id:ebi-a-GCST005068	Vigorous physical activity || id:ebi-a-GCST006098	MR Egger	7	4.55735741	5.39758261	0.43700589
**ebi-a-GCST006098**	ebi-a-GCST005068	LDL cholesterol || id:ebi-a-GCST005068	Vigorous physical activity || id:ebi-a-GCST006098	Weighted median	7	-0.6208293	0.83843447	0.45901939
**ebi-a-GCST006098**	ebi-a-GCST005068	LDL cholesterol || id:ebi-a-GCST005068	Vigorous physical activity || id:ebi-a-GCST006098	Inverse variance weighted	7	-0.2426177	0.66016146	0.71323661
**ebi-a-GCST006098**	ebi-a-GCST005068	LDL cholesterol || id:ebi-a-GCST005068	Vigorous physical activity || id:ebi-a-GCST006098	Simple mode	7	-1.231375	1.53251313	0.45233386
**ebi-a-GCST006098**	ebi-a-GCST005068	LDL cholesterol || id:ebi-a-GCST005068	Vigorous physical activity || id:ebi-a-GCST006098	Weighted mode	7	-1.0821029	1.01739534	0.32843007
**ebi-a-GCST006098**	ieu-a-1050	Ferritin || id:ieu-a-1050	Vigorous physical activity || id:ebi-a-GCST006098	MR Egger	5	-5.2248204	5.49515103	0.41184823
**ebi-a-GCST006098**	ieu-a-1050	Ferritin || id:ieu-a-1050	Vigorous physical activity || id:ebi-a-GCST006098	Weighted median	5	-0.7040971	0.64752149	0.27687222
**ebi-a-GCST006098**	ieu-a-1050	Ferritin || id:ieu-a-1050	Vigorous physical activity || id:ebi-a-GCST006098	Inverse variance weighted	5	-0.1408587	0.76723061	0.85433225
**ebi-a-GCST006098**	ieu-a-1050	Ferritin || id:ieu-a-1050	Vigorous physical activity || id:ebi-a-GCST006098	Simple mode	5	-0.8515134	0.84328604	0.36973273
**ebi-a-GCST006098**	ieu-a-1050	Ferritin || id:ieu-a-1050	Vigorous physical activity || id:ebi-a-GCST006098	Weighted mode	5	-0.9327437	0.76915852	0.29197296
**ebi-a-GCST006098**	ieu-a-302	Triglycerides || id:ieu-a-302	Vigorous physical activity || id:ebi-a-GCST006098	MR Egger	5	-1.4775783	1.33435404	0.34895532
**ebi-a-GCST006098**	ieu-a-302	Triglycerides || id:ieu-a-302	Vigorous physical activity || id:ebi-a-GCST006098	Weighted median	5	-0.1485007	0.26200258	0.5708563
**ebi-a-GCST006098**	ieu-a-302	Triglycerides || id:ieu-a-302	Vigorous physical activity || id:ebi-a-GCST006098	Inverse variance weighted	5	-0.0745059	0.22114262	0.73618224
**ebi-a-GCST006098**	ieu-a-302	Triglycerides || id:ieu-a-302	Vigorous physical activity || id:ebi-a-GCST006098	Simple mode	5	-0.1338663	0.36851473	0.73479489
**ebi-a-GCST006098**	ieu-a-302	Triglycerides || id:ieu-a-302	Vigorous physical activity || id:ebi-a-GCST006098	Weighted mode	5	-0.1563444	0.32885888	0.65928919
**ebi-a-GCST006098**	ukb-b-11349	Folate || id:ukb-b-11349	Vigorous physical activity || id:ebi-a-GCST006098	MR Egger	7	0.44992252	2.05817378	0.83560206
**ebi-a-GCST006098**	ukb-b-11349	Folate || id:ukb-b-11349	Vigorous physical activity || id:ebi-a-GCST006098	Weighted median	7	-0.2016639	0.32005364	0.52863309
**ebi-a-GCST006098**	ukb-b-11349	Folate || id:ukb-b-11349	Vigorous physical activity || id:ebi-a-GCST006098	Inverse variance weighted	7	-0.2577298	0.24461528	0.29206017
**ebi-a-GCST006098**	ukb-b-11349	Folate || id:ukb-b-11349	Vigorous physical activity || id:ebi-a-GCST006098	Simple mode	7	0.0568742	0.46702435	0.90704956
**ebi-a-GCST006098**	ukb-b-11349	Folate || id:ukb-b-11349	Vigorous physical activity || id:ebi-a-GCST006098	Weighted mode	7	0.07601041	0.46533968	0.87561065

**ebi-a-GCST006098**	**ukb-d-30070_irnt**	**Red blood cell (erythrocyte) distribution width || id:ukb-d-30070_irnt**	**Vigorous physical activity || id:ebi-a-GCST006098**	**MR Egger**	**7**	**1.57820562**	**1.79570092**	**0.41969132**
**ebi-a-GCST006098**	ukb-d-30070_irnt	Red blood cell (erythrocyte) distribution width || id:ukb-d-30070_irnt	Vigorous physical activity || id:ebi-a-GCST006098	Weighted median	7	-0.0549663	0.14390626	0.70249175
**ebi-a-GCST006098**	ukb-d-30070_irnt	Red blood cell (erythrocyte) distribution width || id:ukb-d-30070_irnt	Vigorous physical activity || id:ebi-a-GCST006098	Inverse variance weighted	7	-0.2321729	0.21329882	0.27638026
**ebi-a-GCST006098**	ukb-d-30070_irnt	Red blood cell (erythrocyte) distribution width || id:ukb-d-30070_irnt	Vigorous physical activity || id:ebi-a-GCST006098	Simple mode	7	-0.0408618	0.17733468	0.82541927
**ebi-a-GCST006098**	ukb-d-30070_irnt	Red blood cell (erythrocyte) distribution width || id:ukb-d-30070_irnt	Vigorous physical activity || id:ebi-a-GCST006098	Weighted mode	7	-0.0920344	0.18214756	0.63138589
**ebi-a-GCST006098**	ukb-d-30830_irnt	SHBG || id:ukb-d-30830_irnt	Vigorous physical activity || id:ebi-a-GCST006098	MR Egger	7	0.46503747	1.9737129	0.82307752
**ebi-a-GCST006098**	ukb-d-30830_irnt	SHBG || id:ukb-d-30830_irnt	Vigorous physical activity || id:ebi-a-GCST006098	Weighted median	7	0.11870029	0.15191489	0.43459052
**ebi-a-GCST006098**	ukb-d-30830_irnt	SHBG || id:ukb-d-30830_irnt	Vigorous physical activity || id:ebi-a-GCST006098	Inverse variance weighted	7	0.33282495	0.21369539	0.11935806
**ebi-a-GCST006098**	ukb-d-30830_irnt	SHBG || id:ukb-d-30830_irnt	Vigorous physical activity || id:ebi-a-GCST006098	Simple mode	7	0.07719013	0.19009622	0.6987848
**ebi-a-GCST006098**	ukb-d-30830_irnt	SHBG || id:ukb-d-30830_irnt	Vigorous physical activity || id:ebi-a-GCST006098	Weighted mode	7	0.08827653	0.18228932	0.64536599

Tests for horizontal pleiotropy

id.exposure	id.outcome	outcome	exposure	Egger intercept	se	pval
**ebi-a-GCST006098**	ebi-a-GCST005068	LDL cholesterol || id:ebi-a-GCST005068	Vigorous physical activity || id:ebi-a-GCST006098	-0.0463331	0.05171055	0.41130582
**ebi-a-GCST006098**	ieu-a-1050	Ferritin || id:ieu-a-1050	Vigorous physical activity || id:ebi-a-GCST006098	0.04824757	0.05162226	0.41892633
**ebi-a-GCST006098**	ieu-a-302	Triglycerides || id:ieu-a-302	Vigorous physical activity || id:ebi-a-GCST006098	0.01391277	0.01304839	0.36450721
**ebi-a-GCST006098**	ukb-b-11349	Folate || id:ukb-b-11349	Vigorous physical activity || id:ebi-a-GCST006098	-0.0065813	0.01900584	0.74322824
**ebi-a-GCST006098**	ukb-d-30070_irnt	Red blood cell (erythrocyte) distribution width || id:ukb-d-30070_irnt	Vigorous physical activity || id:ebi-a-GCST006098	-0.0168346	0.01658053	0.35653574
**ebi-a-GCST006098**	ukb-d-30830_irnt	SHBG || id:ukb-d-30830_irnt	Vigorous physical activity || id:ebi-a-GCST006098	-0.0012295	0.01822548	0.94882818

S6 Table. 2S-MR results with vigorous physical activity as exposure and lifestyle habits as outcomes

id.exposure	id.outcome	outcome	exposure	method	nsnp	b	se	pval
**ebi-a-GCST006098**	ukb-b-10217	Sweets intake || id:ukb-b-10217	Vigorous physical activity || id:ebi-a-GCST006098	MR Egger	7	-0.0903539	1.5078935	0.95454011
**ebi-a-GCST006098**	ukb-b-10217	Sweets intake || id:ukb-b-10217	Vigorous physical activity || id:ebi-a-GCST006098	Weighted median	7	-0.2367422	0.21224606	0.26467304
**ebi-a-GCST006098**	ukb-b-10217	Sweets intake || id:ukb-b-10217	Vigorous physical activity || id:ebi-a-GCST006098	Inverse variance weighted	7	-0.1760505	0.17921106	0.32592043
**ebi-a-GCST006098**	ukb-b-10217	Sweets intake || id:ukb-b-10217	Vigorous physical activity || id:ebi-a-GCST006098	Simple mode	7	-0.2770321	0.31802813	0.41719033
**ebi-a-GCST006098**	ukb-b-10217	Sweets intake || id:ukb-b-10217	Vigorous physical activity || id:ebi-a-GCST006098	Weighted mode	7	-0.2596458	0.31161898	0.43662814
**ebi-a-GCST006098**	ukb-b-11679	Type of special diet followed: Vegetarian || id:ukb-b-11679	Vigorous physical activity || id:ebi-a-GCST006098	MR Egger	6	0.30989332	0.88272515	0.74325054
**ebi-a-GCST006098**	ukb-b-11679	Type of special diet followed: Vegetarian || id:ukb-b-11679	Vigorous physical activity || id:ebi-a-GCST006098	Weighted median	6	0.02203046	0.0642637	0.73173884
**ebi-a-GCST006098**	ukb-b-11679	Type of special diet followed: Vegetarian || id:ukb-b-11679	Vigorous physical activity || id:ebi-a-GCST006098	Inverse variance weighted	6	0.02308089	0.04874316	0.63584178
**ebi-a-GCST006098**	ukb-b-11679	Type of special diet followed: Vegetarian || id:ukb-b-11679	Vigorous physical activity || id:ebi-a-GCST006098	Simple mode	6	0.02147452	0.10369298	0.84410404
**ebi-a-GCST006098**	ukb-b-11679	Type of special diet followed: Vegetarian || id:ukb-b-11679	Vigorous physical activity || id:ebi-a-GCST006098	Weighted mode	6	0.02451679	0.09506696	0.80677012
**ebi-a-GCST006098**	ukb-b-1996	Salad / raw vegetable intake || id:ukb-b-1996	Vigorous physical activity || id:ebi-a-GCST006098	MR Egger	7	0.66619702	1.2339482	0.61244007
**ebi-a-GCST006098**	ukb-b-1996	Salad / raw vegetable intake || id:ukb-b-1996	Vigorous physical activity || id:ebi-a-GCST006098	Weighted median	7	0.5139604	0.10299379	6.03E-07
**ebi-a-GCST006098**	ukb-b-1996	Salad / raw vegetable intake || id:ukb-b-1996	Vigorous physical activity || id:ebi-a-GCST006098	Inverse variance weighted	7	0.50448517	0.13388668	0.00016456
**ebi-a-GCST006098**	ukb-b-1996	Salad / raw vegetable intake || id:ukb-b-1996	Vigorous physical activity || id:ebi-a-GCST006098	Simple mode	7	0.62702954	0.14130406	0.00438784

**ebi-a-GCST006098**	**ukb-b-1996**	**Salad / raw vegetable intake || id:ukb-b-1996**	**Vigorous physical activity || id:ebi-a-GCST006098**	**Weighted mode**	**7**	**0.58940834**	**0.1467562**	**0.00698862**
**ebi-a-GCST006098**	ukb-b-2209	Oily fish intake || id:ukb-b-2209	Vigorous physical activity || id:ebi-a-GCST006098	MR Egger	7	0.95472369	2.00996591	0.65481403
**ebi-a-GCST006098**	ukb-b-2209	Oily fish intake || id:ukb-b-2209	Vigorous physical activity || id:ebi-a-GCST006098	Weighted median	7	0.53975424	0.15310124	0.00042273
**ebi-a-GCST006098**	ukb-b-2209	Oily fish intake || id:ukb-b-2209	Vigorous physical activity || id:ebi-a-GCST006098	Inverse variance weighted	7	0.48170818	0.21898245	0.02782414
**ebi-a-GCST006098**	ukb-b-2209	Oily fish intake || id:ukb-b-2209	Vigorous physical activity || id:ebi-a-GCST006098	Simple mode	7	0.82403974	0.20581323	0.00708805
**ebi-a-GCST006098**	ukb-b-2209	Oily fish intake || id:ukb-b-2209	Vigorous physical activity || id:ebi-a-GCST006098	Weighted mode	7	0.78663663	0.29210032	0.03590724
**ebi-a-GCST006098**	ukb-b-3881	Fresh fruit intake || id:ukb-b-3881	Vigorous physical activity || id:ebi-a-GCST006098	MR Egger	7	1.30879467	1.11224869	0.29227451
**ebi-a-GCST006098**	ukb-b-3881	Fresh fruit intake || id:ukb-b-3881	Vigorous physical activity || id:ebi-a-GCST006098	Weighted median	7	0.46001672	0.08901784	2.37E-07
**ebi-a-GCST006098**	ukb-b-3881	Fresh fruit intake || id:ukb-b-3881	Vigorous physical activity || id:ebi-a-GCST006098	Inverse variance weighted	7	0.38633487	0.12863774	0.00267088
**ebi-a-GCST006098**	ukb-b-3881	Fresh fruit intake || id:ukb-b-3881	Vigorous physical activity || id:ebi-a-GCST006098	Simple mode	7	0.59733916	0.1169325	0.00220313
**ebi-a-GCST006098**	ukb-b-3881	Fresh fruit intake || id:ukb-b-3881	Vigorous physical activity || id:ebi-a-GCST006098	Weighted mode	7	0.58037912	0.12022717	0.00291812
**ebi-a-GCST006098**	ukb-b-4616	Nap during day || id:ukb-b-4616	Vigorous physical activity || id:ebi-a-GCST006098	MR Egger	7	-1.8168346	0.8575858	0.08766702

**ebi-a-GCST006098**	**ukb-b-4616**	**Nap during day || id:ukb-b-4616**	**Vigorous physical activity || id:ebi-a-GCST006098**	**Weighted median**	**7**	**-0.3203349**	**0.09563435**	**0.00080934**
**ebi-a-GCST006098**	ukb-b-4616	Nap during day || id:ukb-b-4616	Vigorous physical activity || id:ebi-a-GCST006098	Inverse variance weighted	7	-0.1836272	0.12240112	0.1335603
**ebi-a-GCST006098**	ukb-b-4616	Nap during day || id:ukb-b-4616	Vigorous physical activity || id:ebi-a-GCST006098	Simple mode	7	-0.3872459	0.1272748	0.02272693
**ebi-a-GCST006098**	ukb-b-4616	Nap during day || id:ukb-b-4616	Vigorous physical activity || id:ebi-a-GCST006098	Weighted mode	7	-0.3872459	0.11249117	0.01375948
**ebi-a-GCST006098**	ukb-b-6324	Processed meat intake || id:ukb-b-6324	Vigorous physical activity || id:ebi-a-GCST006098	MR Egger	7	0.06859678	1.00946468	0.94845629
**ebi-a-GCST006098**	ukb-b-6324	Processed meat intake || id:ukb-b-6324	Vigorous physical activity || id:ebi-a-GCST006098	Weighted median	7	-0.6292381	0.13853861	5.57E-06
**ebi-a-GCST006098**	ukb-b-6324	Processed meat intake || id:ukb-b-6324	Vigorous physical activity || id:ebi-a-GCST006098	Inverse variance weighted	7	-0.5496216	0.11346205	1.27E-06
**ebi-a-GCST006098**	ukb-b-6324	Processed meat intake || id:ukb-b-6324	Vigorous physical activity || id:ebi-a-GCST006098	Simple mode	7	-0.6879646	0.21816478	0.01972901
**ebi-a-GCST006098**	ukb-b-6324	Processed meat intake || id:ukb-b-6324	Vigorous physical activity || id:ebi-a-GCST006098	Weighted mode	7	-0.6939066	0.21655974	0.01850116

Tests for horizontal pleiotropy

id.exposure	id.outcome	outcome	exposure	Egger intercept	se	pval
**ebi-a-GCST006098**	ukb-b-10217	Sweets intake || id:ukb-b-10217	Vigorous physical activity || id:ebi-a-GCST006098	-0.000797	0.01392445	0.95657282
**ebi-a-GCST006098**	ukb-b-11679	Type of special diet followed: Vegetarian || id:ukb-b-11679	Vigorous physical activity || id:ebi-a-GCST006098	-0.0025594	0.00786299	0.76112246
**ebi-a-GCST006098**	ukb-b-1996	Salad / raw vegetable intake || id:ukb-b-1996	Vigorous physical activity || id:ebi-a-GCST006098	-0.0015039	0.01139436	0.90014308
**ebi-a-GCST006098**	ukb-b-2209	Oily fish intake || id:ukb-b-2209	Vigorous physical activity || id:ebi-a-GCST006098	-0.0043991	0.01856093	0.82205339
**ebi-a-GCST006098**	ukb-b-3881	Fresh fruit intake || id:ukb-b-3881	Vigorous physical activity || id:ebi-a-GCST006098	-0.0085789	0.01027085	0.44163822
**ebi-a-GCST006098**	ukb-b-4616	Nap during day || id:ukb-b-4616	Vigorous physical activity || id:ebi-a-GCST006098	0.01518897	0.00791926	0.11322178
**ebi-a-GCST006098**	ukb-b-6324	Processed meat intake || id:ukb-b-6324	Vigorous physical activity || id:ebi-a-GCST006098	-0.0057496	0.009322	0.56437631

S7 Table. **2S-MR with healthy/unhealthy dietary habits as exposures and vigorous physical activity as outcome to assess reverse causality**

id.exposure	id.outcome	outcome	exposure	method	nsnp	b	se	pval
**ukb-b-1996**	ebi-a-GCST006098	Vigorous physical activity || id:ebi-a-GCST006098	Salad / raw vegetable intake || id:ukb-b-1996	MR Egger	20	0.3848011	0.33578299	0.26681018
**ukb-b-1996**	ebi-a-GCST006098	Vigorous physical activity || id:ebi-a-GCST006098	Salad / raw vegetable intake || id:ukb-b-1996	Weighted median	20	0.27257002	0.06073408	7.19E-06
**ukb-b-1996**	ebi-a-GCST006098	Vigorous physical activity || id:ebi-a-GCST006098	Salad / raw vegetable intake || id:ukb-b-1996	Inverse variance weighted	20	0.31607975	0.0657002	1.50E-06
**ukb-b-1996**	ebi-a-GCST006098	Vigorous physical activity || id:ebi-a-GCST006098	Salad / raw vegetable intake || id:ukb-b-1996	Simple mode	20	0.39372315	0.14841231	0.01570282
**ukb-b-1996**	ebi-a-GCST006098	Vigorous physical activity || id:ebi-a-GCST006098	Salad / raw vegetable intake || id:ukb-b-1996	Weighted mode	20	0.39793054	0.16695029	0.02773826
**ukb-b-6324**	ebi-a-GCST006098	Vigorous physical activity || id:ebi-a-GCST006098	Processed meat intake || id:ukb-b-6324	MR Egger	23	0.13509533	0.17451244	0.44748219
**ukb-b-6324**	ebi-a-GCST006098	Vigorous physical activity || id:ebi-a-GCST006098	Processed meat intake || id:ukb-b-6324	Weighted median	23	-0.0754533	0.03693307	0.04105493
**ukb-b-6324**	ebi-a-GCST006098	Vigorous physical activity || id:ebi-a-GCST006098	Processed meat intake || id:ukb-b-6324	Inverse variance weighted	23	-0.1085666	0.03736846	0.00366899
**ukb-b-6324**	ebi-a-GCST006098	Vigorous physical activity || id:ebi-a-GCST006098	Processed meat intake || id:ukb-b-6324	Simple mode	23	-0.0569462	0.08563101	0.51295016
**ukb-b-6324**	ebi-a-GCST006098	Vigorous physical activity || id:ebi-a-GCST006098	Processed meat intake || id:ukb-b-6324	Weighted mode	23	-0.0357803	0.07544894	0.64000771
**ukb-b-4616**	ebi-a-GCST006098	Vigorous physical activity || id:ebi-a-GCST006098	Nap during day || id:ukb-b-4616	MR Egger	89	-0.1310942	0.09139345	0.15504488
**ukb-b-4616**	ebi-a-GCST006098	Vigorous physical activity || id:ebi-a-GCST006098	Nap during day || id:ukb-b-4616	Weighted median	89	-0.0537114	0.0279262	0.05443828
**ukb-b-4616**	ebi-a-GCST006098	Vigorous physical activity || id:ebi-a-GCST006098	Nap during day || id:ukb-b-4616	Inverse variance weighted	89	-0.0652623	0.02490192	0.00877309
**ukb-b-4616**	ebi-a-GCST006098	Vigorous physical activity || id:ebi-a-GCST006098	Nap during day || id:ukb-b-4616	Simple mode	89	-0.0625663	0.0649349	0.33792525
**ukb-b-4616**	ebi-a-GCST006098	Vigorous physical activity || id:ebi-a-GCST006098	Nap during day || id:ukb-b-4616	Weighted mode	89	-0.0482764	0.05502095	0.38264819
**ukb-b-3881**	ebi-a-GCST006098	Vigorous physical activity || id:ebi-a-GCST006098	Fresh fruit intake || id:ukb-b-3881	MR Egger	53	-0.0277245	0.12616196	0.82694037

**ukb-b-3881**	**ebi-a-GCST006098**	**Vigorous physical activity || id:ebi-a-GCST006098**	**Fresh fruit intake || id:ukb-b-3881**	**Weighted median**	**53**	**0.16136177**	**0.0384663**	**2.73E-05**
**ukb-b-3881**	ebi-a-GCST006098	Vigorous physical activity || id:ebi-a-GCST006098	Fresh fruit intake || id:ukb-b-3881	Inverse variance weighted	53	0.2103584	0.03697867	1.28E-08
**ukb-b-3881**	ebi-a-GCST006098	Vigorous physical activity || id:ebi-a-GCST006098	Fresh fruit intake || id:ukb-b-3881	Simple mode	53	0.16760638	0.09363947	0.07929148
**ukb-b-3881**	ebi-a-GCST006098	Vigorous physical activity || id:ebi-a-GCST006098	Fresh fruit intake || id:ukb-b-3881	Weighted mode	53	0.14738639	0.08743063	0.09783589

Tests for horizontal pleiotropy

id.exposure	id.outcome	outcome	exposure	Egger intercept	se	pval
**ukb-b-1996**	ebi-a-GCST006098	Vigorous physical activity || id:ebi-a-GCST006098	Salad / raw vegetable intake || id:ukb-b-1996	-0.0007564	0.00362056	0.83686113
**ukb-b-6324**	ebi-a-GCST006098	Vigorous physical activity || id:ebi-a-GCST006098	Processed meat intake || id:ukb-b-6324	-0.0037415	0.00262036	0.16803755
**ukb-b-4616**	ebi-a-GCST006098	Vigorous physical activity || id:ebi-a-GCST006098	Nap during day || id:ukb-b-4616	0.00064846	0.00086601	0.45600425
**ukb-b-3881**	ebi-a-GCST006098	Vigorous physical activity || id:ebi-a-GCST006098	Fresh fruit intake || id:ukb-b-3881	0.00227091	0.00115335	0.05440223

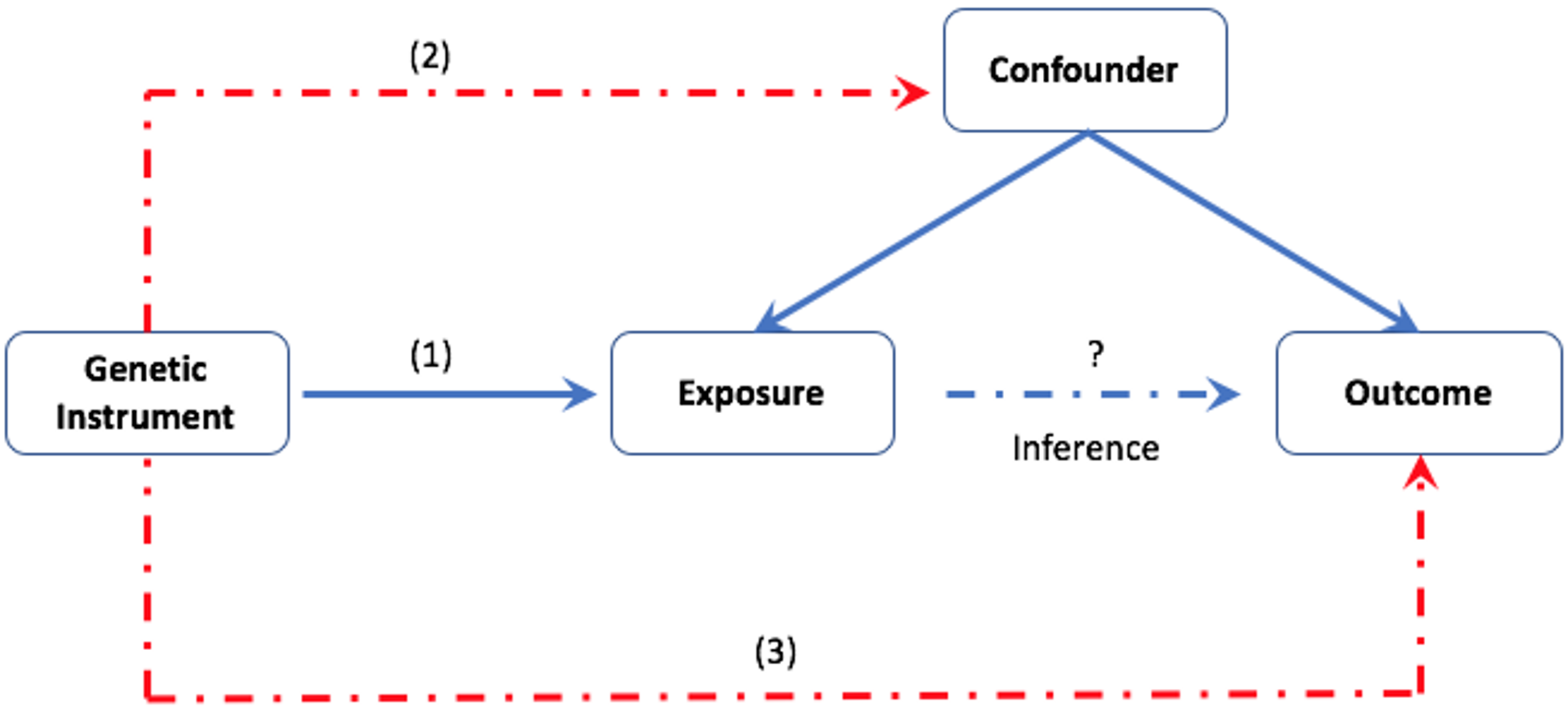

S1 Fig. Assumptions of Mendelian randomization: (1) the genetic instrument is associated with the exposure. (2) the genetic instrument should not associate with a confounder. (3) the genetic instrument should affect the outcome only via the exposure.

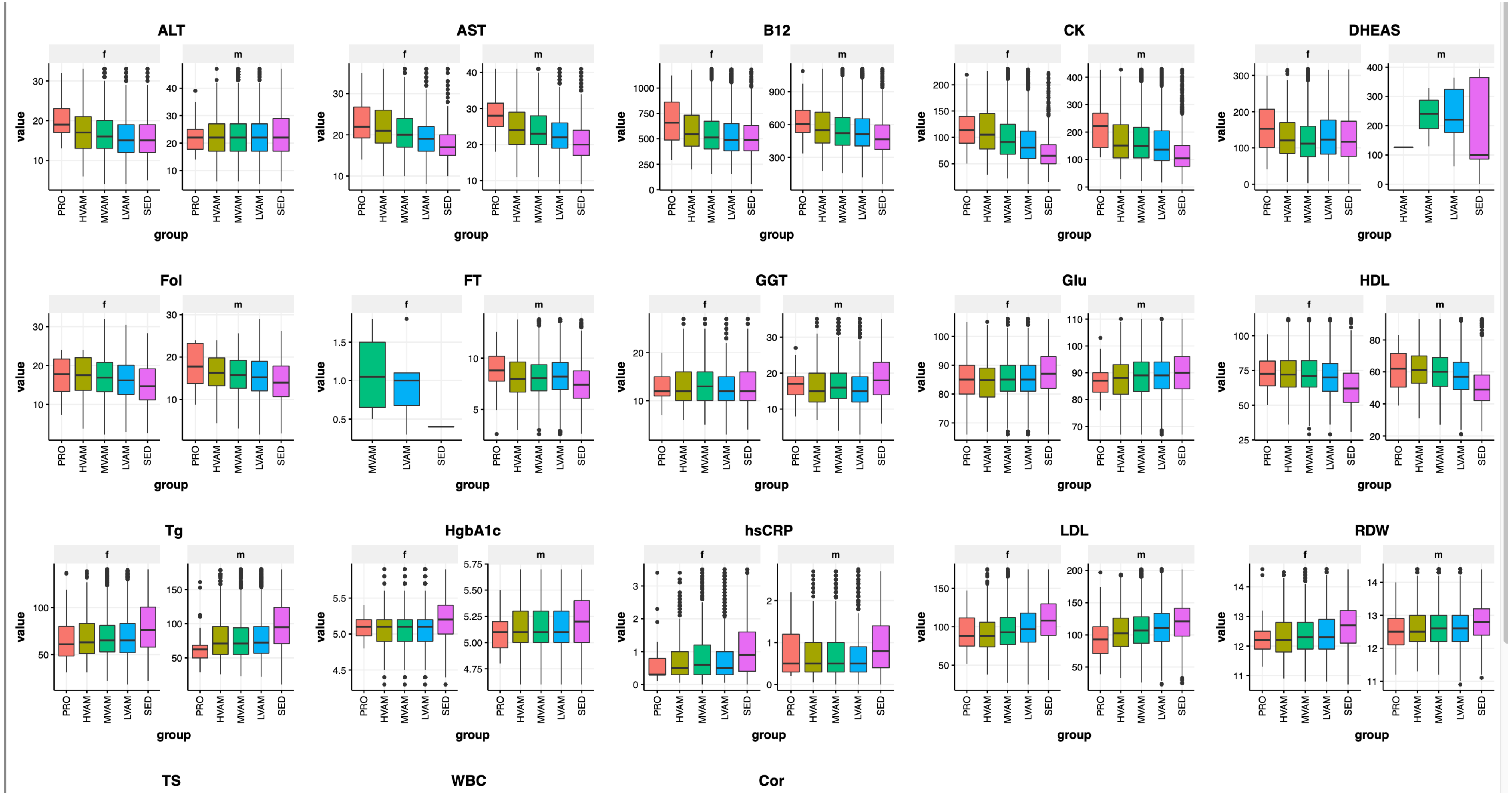

S2 Fig. Blood biomarker levels with respect to self-reported running volume and professional athletes in males (m) and females (f).

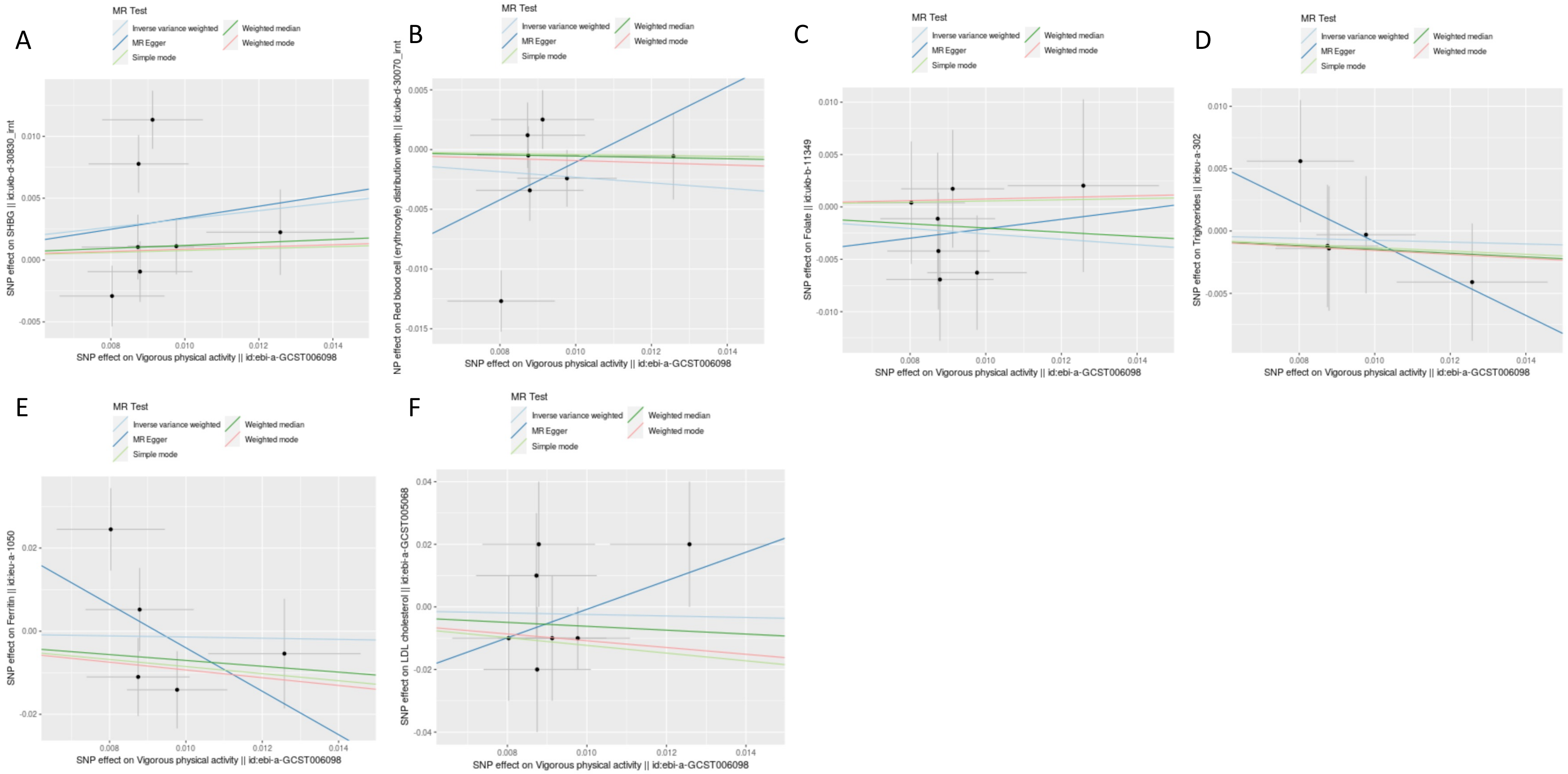

S3 Fig. 2S-MR scatter plot showing effects of vigorous physical activity as the exposure on (A) SHBG (B) Red blood cell count (C) Folate (D) Triglycerides (E) Ferritin (F) LDL as outcomes (see Table 5S for statistical significance).

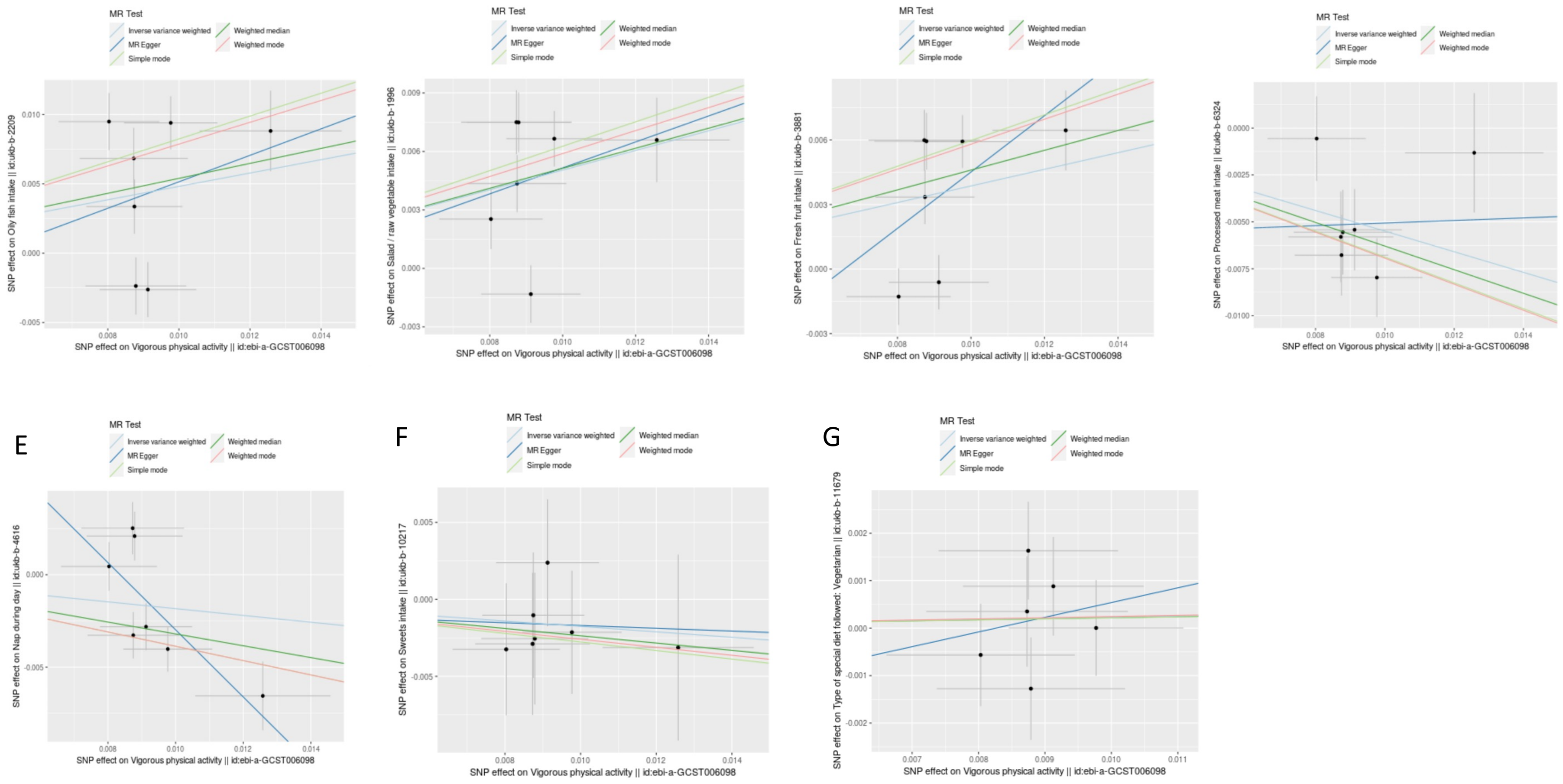

S4 Fig. 2S-MR scatter plot showing effects of vigorous physical activity as the exposure on (A) oily fish consumption (B) salad intake(C) fresh fruit intake (D) processed meat intake (E) daytime napping (F) sweets intake (G) vegetarian diet (see Table 6S for statistical significance).

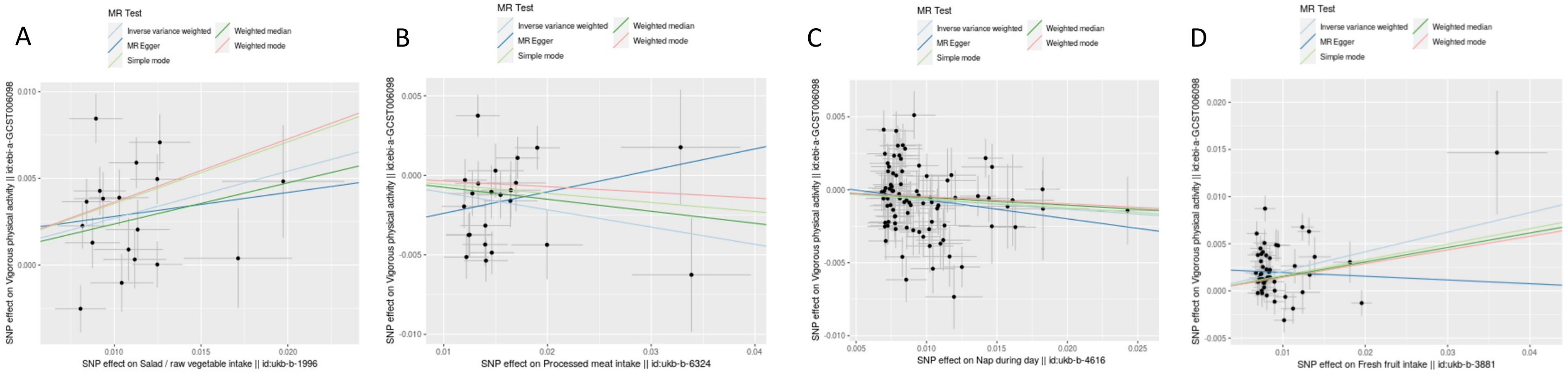

S5 Fig. 2S-MR scatter plot showing effects of dietary behaviors as the exposures on vigorous physical activity: (A) salad intake (B) processed meat intake (C) daytime napping (D) fresh fruit intake (see Table 7S for statistical significance).
