# Supplementary material for: Dose response of running on blood biomarkers of wellness in generally healthy individuals
Source: PLoS One. 2023 Nov 15;18(11):e0293631. doi: 10.1371/journal.pone.0293631 (PMC10651037; doi:10.1371/journal.pone.0293631)
Supplement: Attachment — Submitted filename: PLoS One-Comments (Subir Gupta).pdf [file pone.0293631.s004.pdf]

PONE-D-23-25168

Dose response of running on blood biomarkers of wellness in the generally healthy

Reviewer: Dr. Subir Gupta

Upon a meticulous review of the article in question, I wish to commend the authors for crafting a piece that not only carries immense scientific weight but is also articulated with great clarity. Such insightful work surely merits publication in your distinguished journal. It's admirable how the authors have navigated through a myriad of physiological and biochemical variables (blood biomarkers) across five distinct participant categories and presented their results with lucidity. The experimental framework is robust, the statistical evaluations are apt, and the narrative progresses seamlessly. The references provided are both relevant and adequate. Nevertheless, I'd like to offer a few observations and suggestions:

Original Title: "Dose response of running on blood biomarkers of wellness in the generally healthy."
Proposed Title: "Dose-response relationship between running and blood biomarkers of wellness in generally healthy individuals."

Page 2, Line 8: The mention of "exposure to sunlight" seems somewhat out of context. Could the authors clarify its relevance or indicate if it has been discussed elsewhere in the article?

Page 17, Lines 17-18: The text reads: "These observations suggest that elite endurance runners………to their magnesium status."
Comments: It would be helpful to clarify whether the professional athletes (PRO) participating in this study are specifically elite endurance runners. Kindly integrate this distinction into the main text if accurate.

Page 19, Lines 1-2: The assertion: "Indeed whether exercise………..is inconclusive," needs to be substantiated with a relevant citation.

Table 1: Please include standard deviation (SD) values. I also recommend expressing exercise duration in terms of "h/week" instead of "hr".
