# Supplementary material for: Dose response of running on blood biomarkers of wellness in generally healthy individuals
Source: PLoS One. 2023 Nov 15;18(11):e0293631. doi: 10.1371/journal.pone.0293631 (PMC10651037; doi:10.1371/journal.pone.0293631)
Supplement: Attachment — Submitted filename: Response to Reviwers.docx [file pone.0293631.s005.docx]

**Reviewer #1**: The majority parts of the articles are technically sound. Moreover, the purpose of the study is very sound since it focused on healthy active population. Among the few drawbacks of the study the way the study subjects categorized into groups based on the duration of the activity (>10hours per week and less that 10hours per week is not appropriate. Moreover, the reliability/validity of the information sources in relation to the biomarker tests and lifestyle habits of the study subjects didn't consider the immediate effects of medical services and medication conditions of the respondents at the time of reporting the volume of exercise and biomarker test results. Medical services and lifestyle habits specially all the habits in addition to exercises/running are very important to reach informative decision in this research. So, the above two points need further explanation or modification.

**Response to Reviewer #1:** We appreciate the reviewer's feedback and are pleased that they find the majority of our study technically sound and recognize the importance of our focus on a healthy, active population. We also appreciate the reviewer pointing out an opportunity to improve the clarity around our experimental design as it pertains subject groupings.

Regarding the categorization of study subjects, we want to clarify that we actually categorized them into five groups. These groups include professional endurance runners, high volume amateur runners (>10 hours per week), medium volume amateur runners (3-10 hours per week), low volume amateur runners (<3 hours per week), and the sedentary. We now added a sentence starting on line 125 the explicitly states this categorization (“The cohort was divided into five groups:…”). These groupings were determined based on the respondents' self-reported data.

We acknowledge the potential influence of medication use on our analysis, and we now address it starting on line 461 (“These factors, such as diet, sleep, and/or medications were not readily ascertained in this free-living cohort…”) and in the Study Limitations section (line 595). We noted that unmeasured confounders such as medications, nutritional supplements, and unreported health conditions may exist. However, given the nature of our cohort, which primarily consists of self-selected, generally healthy individuals, the impact of significant medication use is expected to be limited. We believe that the observed trends in healthier biomarker levels with increased reported running volume support this assertion.

Furthermore, we recognize the importance of lifestyle habits beyond exercise in influencing our results. To address this, we employed statistical genomics, specifically two-sample Mendelian randomization with physical activity as the exposure. This analysis allowed us to explore other potential habits and behaviors contributing to improved biomarker signatures in physically active runners within our cohort. We kindly refer the reviewer to the "Vigorous physical activity associates with healthier behaviors" section in the results for a detailed examination of this aspect. Notably, our entire cohort is composed of health-conscious individuals within the same health advisory platform, with the primary differentiator being self-reported running activity. We also controlled for key variables such as age, sex, and BMI in our ANOVA analyses.

We hope these explanations clarify our approach and address the reviewer's concerns adequately.

**Reviewer #2**: How your data is reliable by using A cross-sectional study design? &

How again the Data is reliable by using self-reported running. I understand that Biomarkers are objective measure, but do you think that Self-report is trustworthy? Thank you

**Response to Reviewer #2:** We appreciate the reviewer's questions and concerns regarding the reliability of our runners data, which is largely derived from self-reported exercise habits. Cross-sectional studies inherently have limitations when it comes to establishing causality, and we acknowledge this challenge. To address potential confounding factors, we conducted additional causal analyses, specifically investigating the effects of BMI on the biomarkers under examination to begin to disentangle the relative contributions of known factors. Furthermore, we performed secondary Mendelian randomization (MR) analyses to identify and account for potential confounders in our findings. We kindly invite the reviewer to explore the "Vigorous physical activity associates with healthier behaviors" section in the results for a comprehensive exploration of these confounding aspects.

Regarding the reliability of self-reported running activity, we recognize that self-reports can be subject to biases, and individuals may tend to overestimate their exercise commitment. To address this drawback, we added language addressing these limitations in the “Study limitations” section (Line 579: “First, it is generally known that subjects tend to overestimate their commitment to exercise …”). We do note that our study cohort comprises self-selected individuals who are health-conscious and possibly less prone to over-report their running volume. Additionally, the robust increasing trend in baseline levels of muscle damage biomarkers (CK, AST), which are known to be associated with participation in sports and exercise, provides indirect evidence that the different running groups in our study were indeed engaging in increasing volumes of strenuous physical activity.

While self-reporting has its limitations, it remains a valuable method for capturing individuals' exercise behaviors in large-scale observational studies. We took measures to mitigate potential biases, and our findings align with established trends in biomarker responses to physical activity.

**Reviewer #3**: Upon a meticulous review of the article in question, I wish to commend the authors for crafting a piece that not only carries immense scientific weight but is also articulated with great clarity. Such insightful work surely merits publication in your distinguished journal. It's admirable how the authors have navigated through a myriad of physiological and biochemical variables (blood biomarkers) across five distinct participant categories and presented their results with lucidity. The experimental framework is robust, the statistical evaluations are apt, and the narrative progresses seamlessly. The references provided are both relevant and adequate. Nevertheless, I'd like to offer a few observations and suggestions:

**Response**: We appreciate the reviewer's positive feedback and kind words about our manuscript. We eagerly await their observations and suggestions should they see further opportunities to improve our work based on our responses to the current suggestions.

Original Title: “Dose response of running on blood biomarkers of wellness in the generally healthy.”

Proposed Title: “Dose-response relationship between running and blood biomarkers of wellness in generally healthy individuals.”

**Response**: Thank you – title has been changed.

Page 2, Line 8: The mention of “exposure to sunlight” seems somewhat out of context. Could the authors clarify its relevance or indicate if it has been discussed elsewhere in the article?

**Response**: Thank you for the suggestion, we removed this as we agree it was not relevant in this manuscript.

Page 17, Lines 17-18: The text reads: "These observations suggest that elite endurance runners………to their magnesium status."

Comments: It would be helpful to clarify whether the professional athletes (PRO) participating in this study are specifically elite endurance runners. Kindly integrate this distinction into the main text if accurate.

**Response**: Thank you for the clarifying suggestion. We included the pro/elite endurance runners clarification within the abstract as well as a section heading (lines 7 and 425)

Page 19, Lines 1-2: The assertion: “Indeed whether exercise………..is inconclusive,” needs to be substantiated with a relevant citation.

**Response**: Thank you – citations have been added.

Table 1: Please include standard deviation (SD) values. I also recommend expressing exercise duration in terms of "h/week" instead of "hr".

**Response**: Thank you for the catch – units changed to “h/week” and SDs added to Table 1.

We are grateful for your valuable feedback, which has contributed to improving the clarity and accuracy of our manuscript.
